# Improving detection of work-related asthma: a review of gaps in awareness, reporting and knowledge translation

**DOI:** 10.1186/s13223-020-00470-w

**Published:** 2020-08-06

**Authors:** Madison MacKinnon, Teresa To, Clare Ramsey, Catherine Lemière, M. Diane Lougheed

**Affiliations:** 1 Asthma Research Unit, Kingston Health Sciences Centre, 72 Stuart Street, Kingston, ON K7L 2V7 Canada; 2 Division of Respirology, Department of Medicine, Queen’s University, 102 Stuart Street, Kingston, ON K7L 2V6 Canada; 3 The Hospital for Sick Children, Research Institute, Dalla Lana School of Public Health, University of Toronto, 686 Bay St, Toronto, ON Canada; 4 Department of Medicine, Rady Faculty of Health Sciences, University of Manitoba, 810 Sherbrook St., Winnipeg, MB R3A1R9 Canada; 5 Department of Chest Medicine, CIUSSS du nord de l’île de Montréal, Hôpital du Sacré-Cœur de Montréal, 5400 Gouin West, Montreal, QC H4J 1C5 Canada

**Keywords:** Asthma, Work-related asthma, Occupational asthma, Work-exacerbated asthma, Work-aggravated asthma, Screening, Diagnosis, Knowledge translation, Implementation

## Abstract

**Background:**

Work-related asthma (WRA) accounts for up to 25% of all adults with asthma. Early diagnosis is key for optimal management as delays in diagnosis are associated with worse outcomes. However, WRA is significantly underreported and the median time to diagnosis is 4 years. The objective of this review is to identify the gaps in awareness and reporting of WRA and identify gaps in current knowledge translation strategies for chronic disease in general, and asthma specifically. This will identify reasons for delays in WRA diagnosis, as well inform suggestions to improve knowledge translation strategies for dissemination and implementation of WRA prevention and management guidelines.

**Methods:**

Non-systematic literature reviews were conducted on PubMed with a focus on work-related asthma screening and diagnosis, and knowledge translation or translational medicine research in asthma and chronic disease. In total, 3571 titles and abstracts were reviewed with no restriction on date published. Of those, 207 were relevant and fully read. Another 37 articles were included and reviewed after citation reviews of articles from the initial search and from suggestions from editors. In total, 63 articles were included in the final review.

**Results:**

Patients, employers, and healthcare professionals lack awareness and under-report WRA which contribute to the delayed diagnosis of WRA, primarily through lack of education, stigma associated with WRA, and lack of awareness and screening in primary care. Knowledge translation strategies for asthma research typically involve the creation of guidelines for diagnosis of the disease, asthma care plans and tools for education and management. While there are some prevention programs in place for certain industries, gaps in knowledge translation strategies including lack of screening tools currently available for WRA, poor education of employers and physicians in identifying WRA, and education of patients is often done post-diagnosis and focuses on management rather than prevention or screening.

**Conclusion:**

Future knowledge translation strategies should focus on educating employees and employers well before potential exposure to agents associated with WRA and screening for WRA in primary care to enable health care providers to recognize and diagnose WRA.

## Background

**Fig. 1:**
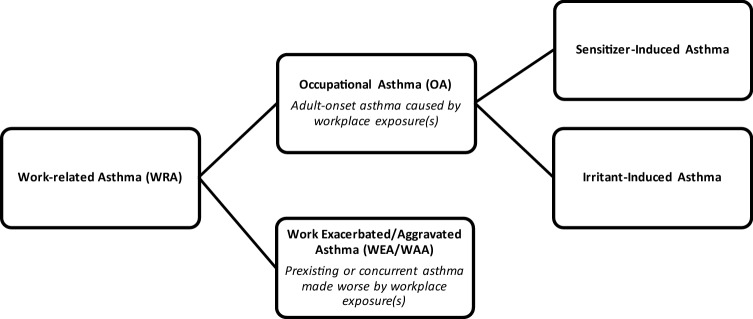
Classification of Work-related Asthma [1]

Work-related asthma (WRA) is a term that encompasses two types of asthma: occupational asthma (OA) which describes asthma induced by a sensitizer or irritant in the workplace or work-exacerbated asthma (WEA), also known as work-aggravated asthma (WAA), which describes preexisting or concurrent asthma made worse by workplace exposure(s) (Fig. 1) [1]. It is estimated that WRA accounts for up to 25% of all adults with asthma in the population [2]. Industries that have a high prevalence of WRA include educational services, health services, manufacturing, public administration and agriculture. The most common occupations to have workers with WRA are teachers, farm and construction workers, administrators or managers, and cleaners [3].

Workers who are continuously exposed to WRA triggers have significant morbidity, are more likely to have uncontrolled asthma, and display absenteeism and presenteeism, a loss of productivity at work due to illness [4–6]. Those burdened with WRA are significantly more likely to be unemployed, be temporarily unable to work due to their symptoms, and take more sick days than asthmatics without WRA [7]. Patients with WRA are also more likely to have depression and a poorer quality of life than those not burdened with the disease [8, 9]. Subjects with WRA use more medical resources and have more visits to their family doctor, the emergency department and hospitalizations than those not affected, which increase healthcare costs [10, 11]. Workers face a financial burden from the disease and many experience financial barriers, such as loss of income from missing work due to asthma symptoms [12, 13]. Employers can be required to provide compensation for their employees, often through compensation boards or the government, so employers and taxpayers are also financially affected by WRA [14, 15].

Therefore, early detection and diagnosis of WRA is critical for good management and reducing the burden and morbidity of WRA, as delayed diagnosis is strongly associated with worse outcomes [4]. Unfortunately, WRA is severely under-diagnosed and under-reported and the median time to diagnosis of WRA is 4 years after symptoms first appear [13, 16].

Knowledge translation is a term that describes implementing research findings into practice [17]. There are many proposed strategies for knowledge translation, however successful implementation of such strategies and proposals is difficult. Implementation has been called the “Achilles heel” of innovation and is often the limiting factor to successful knowledge translation [17]. Graham et al. [18], attempted to organize this process by creating the Knowledge to Action (KTA) Framework to guide knowledge translation by highlighting key steps and elements in this process.

The KTA process has two phases: Knowledge Creation and Knowledge Action. Knowledge Creation identifies the types of research found in healthcare and organizes it into three levels.: Knowledge Inquiry, the least synthesized research available including primary studies, Knowledge Synthesis, which includes more refined research such as systematic reviews, meta-analyses and syntheses, and finally, Knowledge Tools/Products, the most synthesized tools and research, designed to deliver knowledge in a concise, easy-to-understand way. [18]

The Knowledge Action phase occurs after or sequentially during Knowledge Creation. Knowledge Action is a cycle that identifies eight key steps for implementation [18]. Briefly, the steps are: identifying a problem that needs addressing, reviewing the research available to identify gaps between knowledge and practice, adapt the knowledge and tailor it to the research question, identify the barriers that could affect the use of the knowledge, select, tailor and implement interventions, monitor the intervention post-implementation to see the effects, evaluate the outcomes to determine the impact of the implementation, and assess the barriers to sustainability to promote further use of the research [18].

Chronic conditions, such as asthma, have a high burden on the workplace, greatly affect patient lifestyle and require constant management, so dissemination of research in a way that can educate the public and patients should be a primary objective for researchers [19, 20]. However, knowledge gaps exist in patients with chronic diseases showing a need for more education [21].

Therefore, the objective of this review is to identify the gaps in awareness and reporting of WRA which contribute to the delayed diagnosis by identifying who is under-reporting WRA and why, and what is impeding awareness of WRA in the workplace and healthcare. As well, we aim to identify gaps in current knowledge translation strategies for asthma and chronic disease, and to see if they are successfully targeting and educating those affected by WRA. Finally, we will suggest knowledge translation strategies to address gaps in the assessment and timely diagnosis of WRA.

**Table 1:** Summary of gaps, key findings and future steps

Gap to explore	Question to address gap	Key findings	Key messages and future steps
Lack of Awareness of WRA	Who lacks awareness of WRA? What are the knowledge gaps?	Patients, employers and physicians lack knowledge and awareness of potential asthma triggers and workplace-symptom relationships	Increase education of workplace exposures and their relationship to asthma symptoms Improve screening for WRA
Under-reporting of WRA	Who is under-reporting WRA and why is this happening?	Employees fear stigma from employers if symptoms or concerns are expressed Employee-employer relationships affect employees’ decisions to report Physicians report lack of time, awareness and access to specialists as barriers to reporting	Encourage discussion of workplace triggers between employer and employees Enable detailed occupational history between health care providers and patients Improve access to specialists and objective testing
Gaps in current KT strategies	Are current KT strategies successful in targeting and educating those affected by WRA?	Most KT strategies focus on asthma management and education post-diagnosis Paucity of tools for screening Limited worker/patient education prior to potential exposure(s)	Focus KT strategies on effective education of workers and employers regarding potentially hazardous workplace exposures and development and implementation of effective screening tools for diagnosis

*KT* knowledge translation, *WRA* work-related asthma

## Methods

Three non-systematic literature searches were conducted on PubMed. The first included key words: “work-related asthma”, “occupational asthma”, “work-exacerbated asthma” and “diagnosis”. The search criteria included original articles, human species and had no date restriction. Two other literature reviews with keywords “knowledge translation” or “translational medicine research” for chronic disease and asthma were conducted with the same criteria as above. Both keywords were applied to obtain a cohesive search of all potential knowledge translation strategies. While “translational medicine research” is a broad term that encompasses research from molecular biology to clinical trials, the authors ensured only articles that specifically mentioned potential knowledge translation strategies were included. In total, the searches returned 3571 articles, and those titles and abstracts were reviewed. Articles were excluded on the basis of not being in English, focusing on child or adult non work-related asthma and not focusing on human species. Of those, 207 were relevant and fully read. An additional 37 articles were included from citation reviews of the articles from the initial search and from feedback from editors. In total, 63 articles were included in the final review, with most of the 207 articles excluded due to repeating information.

## Results

### Gaps in awareness and reporting of WRA

The literature review identified three groups of people that contribute to the gaps in reporting and awareness of WRA: patients, employers and physicians, both primary care and specialists. Barriers impacting their awareness and reporting of WRA are discussed by group below.

#### Patients

Patients with WRA face many challenges in managing and reporting suspected WRA, one being that they are unable to identify potential exposures that could cause WRA [13]. Santos et al. reports that only around one-third of employees with OA had previous knowledge that exposure to certain work agents could induce asthma. The workers who were knowledgeable of the effects of work agents on their asthma stated that this knowledge influenced their decision to see a doctor earlier, while most workers only went to see a doctor when their symptoms became unbearable [13]. Similarly, in a study looking at workers with suspected or diagnosed OA, 25% of workers with OA did not recognize the onset of their symptoms, 45% of workers thought that the asthma symptoms they were experiencing were normal, 40 percent of workers ignored their symptoms, and 30% of workers only suspected their workplace was related to their asthma when a serious illness struck them [22]. Another study reported that even though workers noticed their symptoms worsened during the work week and work shifts, they were often still working in the same job, causing exacerbation of their asthma and increasing morbidity [5].

Patients who are aware of their symptoms and the effect of the workplace on their asthma identified other reasons for why they delayed visiting a doctor or reporting exacerbations. Despite having productivity loss at work and increased morbidity, workers avoid calling in sick or taking days off for fear of judgment or stigma from their coworkers [4]. This stigma is further evident as workers are hesitant to report health hazards or request assessments of their workplace for fear of losing their job [23]. Some even avoided a diagnosis for fear it would require them to change jobs [13]. Workers do not discuss their symptoms with fellow employees or their management for fear of job loss or an effect on their income level [22].

The healthy worker effect could influence this delayed diagnosis as well. The healthy worker effect describes employees who leave, change or choose jobs that reduce their symptoms, improve asthma management or reduce the chance of an asthma attack [24]. Thus, this could contribute to the underreporting of WRA because employees with WRA are more likely to quit or alter job duties as a way to cope with their asthma, and nothing is done to change the workplace itself [23, 25]. Conversely, some patients experience reporter fatigue for their symptoms, which could also instigate the healthy worker effect as they leave the workplace because they feel their concerns are not being heard [26].

##### WEA versus OA

There is a discrepancy in the awareness and diagnosis of WEA and OA. The median waiting time for diagnosis is shorter with WEA patients than OA patients, and OA patients were more likely to change jobs, have more income loss and worse quality of life [13, 27, 28]. WEA patients are more likely to realize their symptoms were work-related, keep their job, and consult a physician than OA patients [13, 27]. Since some WEA patients have preexisting asthma, they might already know or have previously received education on how to manage asthma. Alternatively, it is possible that since they are knowledgeable of asthma symptoms they can make the connection to their workplace earlier, thus reducing exposure and consulting a physician earlier [1].

#### Employers

Employers and their relationship with their employees can impact the diagnosis of WRA [22]. Poor relationships between employees and their employers over health matters have been described, with employees claiming managers were ignorant or dismissive of their health matters or more focused on the success of the company rather than the health of workers [22, 29]. In one study, 85% of workers described having poor relationships with their managers in terms of health matters and often did not feel encouraged to report health issues for fear of losing their job [22]. Therefore the employees might stay in their position and continue to be exposed to triggers of their asthma to avoid the potential financial downfall if they are subsequently let go [22].

Employers often have limited knowledge of potential triggers in their workplace, which leads to limited healthcare options for employees. In a study surveying the perception of workers with WRA on the follow-up healthcare they received, only 6.5% of workers said that a physician assessed their workplace, and half of those said the physician that completed the assessment was an occupational health specialist [23]. In a study looking at the route people with potential OA took to secondary care, they found the occupational health options available to be variable between the workers [30]. Most of the workers had access to some type of occupational healthcare, but only a little more than half had this service available to them full time. As well, health surveillance was not an option for all workers and very few had an annual health assessment [30]. Finally, few workers have access to workplace health-and-safety programs, which limits their education and awareness of the harmful substances in their workplace [13].

#### Healthcare practitioners

##### Primary care

Large gaps in knowledge exist in asthma care, management and diagnosis in primary care [31]. Physicians often do not take accurate or detailed workplace histories of their patients which would help identify the link between work exposures and asthma symptoms [5, 25, 32]. Furthermore, few patients are told that their asthma is work-related [5, 33]. This has an effect on the diagnosis and morbidity of patients, as one study surveying workers exposed to welding fumes found that many workers experience symptoms for a long time before receiving their diagnosis [5]. One-third of those workers who were diagnosed did not know they had asthma, and of those with a diagnosis, only two-thirds were told by their physician that their asthma was work-related [5]. Similarly, in a study looking at the prevalence of WRA in the USA, 39% of the patients with lifetime asthma reported work-related exacerbations of asthma at their current or previous job, but only 10.5 to 13.5% of workers were told that their asthma was work related [33]. Patients report having to consult their primary care physicians multiple times, sometimes more than five, before a referral to a specialist is made for their symptoms [29]. Physicians have cited that barriers to referring patients to a specialist include a lack of access to a specialist or simply lack of timely access to one [32]. Both factors can be detrimental to asthma diagnosis and management because primary care physicians are one of the first healthcare practitioners consulted by people experiencing asthma symptoms [13]. These delays have a major impact on asthma management and morbidity and could potentially increase the number of hospitalizations of workers [5].

There are gaps and barriers in the implementation of asthma care guidelines in primary care [34]. An important gap in knowledge is the failure of physicians in using an objective measure, like spirometry, to diagnose asthma [35]. Stanbrook et al., claim that almost half of patients with physician-diagnosed asthma have not completed spirometry, which potentially makes their clinical diagnosis incorrect, as the patients have not undergone any test that specifically distinguishes asthma from other similar conditions. Thus, asthma care guidelines are not implemented as the patients could be misdiagnosed. One study found that some primary care physicians do not implement asthma guideline recommendations when diagnosing asthma in patients, including failing to write a management plan or a referral for asthma education [34]. About half of these physicians said they did not know or failed to remember to talk about their patient’s concerns. These gaps in knowledge have in part been explained by barriers these physicians face like lack of time and resources, especially access to objective tests, they add to the lack of knowledge patients have regarding their asthma management [34, 35].

##### Specialists

Those who present with asthma are often referred to specialists, but specialists can also have difficulty screening and identifying WRA [36, 37]. Specialists fail to ask if symptoms of asthma are work-related, and in one study looking at how occupational aetiology was considered when people with asthma symptoms presented to the emergency department or an acute medical unit, only one-fifth of people were asked about their employment status [38]. Specialists can also be unknowingly biased in their diagnosis of WRA [39]. Specialists tend to diagnose WRA when it is triggered by an irritant or in a workplace with which they are familiar, which can cause them to miss potential irritants of their patient’s WRA [36, 39]. Clinics that have occupational specialists or patient representatives who assess their ability to return to work and facilitate successful outcomes between workers and employers had patients with less depression, less anxiety about the workplace and fewer mood disturbances than clinics without workplace specialists [40]. Occupational hygienists sometimes identify potential sensitizers of patient’s OA that clinicians missed during their screening, so they can be very beneficial to the diagnosis of WRA and this displays the difficulty specialists can have in identifying WRA [36].

##### Communication and time

Two other factors that seem to contribute to the delays in diagnosis and poor education of WRA are communication between doctors and patients and the time constraints of physicians. A significant number of people fail to mention the potential relationship between their work and asthma symptoms to doctors, and only one in seven asthma patients communicate their workplace might induce their asthma to doctors [41, 42]. This could be due to multiple factors, such as the patients not being aware themselves of the workplace irritants, fear of diagnosis for causing job loss or impact their income, and fear of stigma from the employer [4, 13, 24, 40]. Additionally, a fair proportion of subjects with work-related asthma have delayed isolated reactions to offending agents. This causes a delayed recovery for the subjects and they may miss the association of the workplace with their asthma symptoms [43]. Finally, this could occur because doctors do not take in-depth workplace histories, thus not prompting a discussion of the workplace with their patients [5, 25].

Physicians state limited time in clinic affects how they screen their patients and that there is not enough time to conduct a complete workplace history [32, 39]. Physicians have cited lack of time as the main barrier for implementing asthma care guidelines as well [34]. A study found that physicians often fail to provide written action plans for their patients and reported that though 90% of physicians said they were aware of the asthma guidelines, only 6% of primary and secondary care specialists said they always used them, and around 30% said they used them “most of the time” [44].

### Gaps in knowledge translation for WRA

The literature review returned knowledge translation strategies within the Knowledge Creation phase of the KTA cycle. These includes a multitude of primary studies on WRA, which would fall under “Knowledge Inquiry”, and could be considered an “unrefined, “unmanageable” amount of information and studies that cannot be easily accessed by all [18]. Therefore, it can be difficult for this research to be translated to the public. However, McElfish *et al.* used a community-based research process to set an agenda for health research that showed involving the public on research initiatives is suggested as a means of ensuring the research, even primary studies, leads to change [45]. The researchers engaged community stakeholders who identified three areas of research they felt were major issues (all of which happened to involve chronic disease), and research protocols and action steps were written. Through this, ten grant proposals were submitted, five of which received funding which lead to publications and presentations [45].

The literature search also returned strategies that fall under the next phase in the Knowledge Creation, termed Knowledge Synthesis. This includes the common strategy of summarizing research through the publication of evidence-based clinical practice guidelines. However, guideline implementation can be difficult or fail if researchers do not properly identify patient or clinical needs [46], which has been an issue for physicians treating patients with asthma who cite many difficulties or barriers to implementing asthma clinical guidelines, as previously discussed [34, 44]. One successful example of implementation occurred in Australia, where a research team identified a gap between evidence and practice in chronic disease management [47]. They created evidence-based resources such as systematic reviews and summaries that clinicians could access for treatment and management of their patients with chronic disease. In 4 years, this team created over one hundred summaries and twenty-six recommended practices for chronic disease management. Further, clinicians have been using these resources to guide their practice and it has made clinicians audit their practice to see if it is in fact evidence-based [47].

Other current knowledge translation strategies actively being used for asthma treatment and management fall under the final stage of Knowledge Creation: Knowledge Tools or Products and have been further categorized into similar categories.

#### Prevention programs in industry

Medical surveillance programs of workplaces for sensitizers that could be encountered are a general prevention program found in industries that are at risk for work-related asthma [48]. Some high-risk industries have implemented prevention or surveillance programs. For example, there is a program called the Coal Worker’s Health Surveillance Program (CWHSP) put in place for the safety of coal workers to detect respiratory disease early [49]. The CWHSP offers chest radiographs as well as spirometry and health assessment questionnaires to coal miners in an attempt to detect potential lung disease early. This has been expanded and is in an early implementation phase and it is mandatory for all coal miners be take part in this program [49].

Another example is the textile industry in the USA. The Occupational Safety and Health Administration (OSHA) has rules and regulations in regard to those workers exposed to cotton dust. There are exposure limits for an 8 h day, and they have dust control measures such as vacuuming floors, supplying respirators for employees, providing free annual medical exams and trainings [50].

#### Self-reporting, self-management and education tools

Many knowledge translation strategies for asthma have focused on improving how patients self-report their symptoms. In as early as 1993, serial peak expiratory flow (PEF) monitoring and patient symptom diaries were compared to see if the diary could identify exacerbations similar to the PEF recordings [51]. Both the symptom diary and PEF recordings were able to detect asthma exacerbations. While the sensitivity and specificity of the diary were not assessed, this study showed that self-reporting symptoms could be useful in identifying exacerbations [51].

Self-management plans are frequently used to facilitate patient education and management. “Asthma eBooks” with information on the disease and management have been created for adults with asthma and parents of children with asthma [52]. An asthma self-management app has been designed to promote self-management behaviours in adolescents by including a diary, reminders, a place for them to document triggers, and information for them to identify their asthma severity [53, 54]. Home visit programs have also been implemented for families in low-income areas. An educator provides the family with information on asthma and assesses their home for potential triggers to help the family adjust to this new diagnosis and promote better management [55]. For WRA specifically, those with WRA are more likely to have received some form of education for their asthma management, most often in the form of written asthma plans which are strongly recommended for those with asthma [56].

Another strategy is to improve the communication of research findings with the public and patients, particularly through disseminating the research into words, actions or items the public can understand. One example is the Boot Camp Translation process, created by a community-based council in Colorado [57]. Simply, this process works to rewrite research into messages and statements that are easy to understand by the public [57]. Often this process will lead to guidelines being created or increase awareness of prevention or management of diseases. The Boot Camp Translation Process applied to asthma diagnosis as well. Asthma care guidelines were disseminated into an “Asthma toolkit” that contained tools for healthcare providers and patients for diagnosis and management of disease [57].

One study looked at the effect of combining asthma education and management plans in one through the Primary Care Asthma Pilot Project (PCAPP) [58]. The PCAPP evaluated a primary care asthma program to see how it affected patient outcomes after patients were on this plan for 1 year. The PCAPP included five components for patients with asthma: an asthma care map created by the Ontario Thoracic Society, a treatment flow chart, program standards, a written action plan for patients and core asthma education. The PCAPP led to patients having better control of symptoms, fewer exacerbations, less healthcare utilization and reduced productivity loss [58].

#### Asthma care pathway

An asthma care pathway for adults in the emergency department called the Emergency Department Asthma Care Pathway (EDACP) was created to guide emergency physicians in their care of asthma and ensure patient education [59]. Clinicians had a positive response to this. They found it facilitated patient discussion, guided care decisions and was considered useful in managing asthma in the emergency department, but the research team did find some barriers with implementation [59]. Physicians cited lack of training and time to complete the EDACP as barriers, which is a similar barrier claimed by primary care physicians in the delay in diagnosing asthma [34, 59].

#### Questionnaires

Questionnaires are inexpensive, easily distributed tools that are often used to gather information on symptoms and exacerbations for asthma [60]. Three questionnaires created specifically for WRA were found, two of which are designed for screening for potential WRA [60–62]. One questionnaire was created to investigate the causes and frequency of exacerbation of WRA through subjects monitoring their PEF at home and work while self-reporting their symptoms. However, when this was validated against serial monitoring of PEF, the questionnaire lacked sensitivity which limits its use in care [60].

The Occupational Asthma Screening Questionnaire–11 Items (OASQ-11), was developed as part of a surveillance program to screen for OA in workers [61]. When the OASQ-11 was evaluated in a clinic for those referred for suspected OA it accurately identified 80% of workers with suspected OA, and identified wheezing at work to be the symptom with highest negative predictive value. However, further validation is required before it can be implemented [61].

Another self-administered screening questionnaire called the Work-related Asthma Screening Questionnaire-Long Version (WRASQ(L)) was developed for use in primary care to improve the screening and recognition of potential WRA [62]. The initial assessment of the WRASQ(L) determined it had content and face validity, good test re-test ability and a low respondent burden, but requires validation against an objective test for WRA to justify its implementation in the workplace [62].

## Discussion

This review has identified three target groups that contribute to the gaps in awareness and reporting of WRA which contribute to a delayed diagnosis. Lack of awareness of WRA is seen in patients, employers and healthcare professionals, with patients and healthcare workers under-reporting the disease as well.

Many patients lack awareness and recognition of the onset of asthma and the potential triggers in the workplace, indicating a severe gap in education of workers [13]. The discrepancy in awareness, education and hospital visits between WEA and OA patients emphasize this. Some WEA patients have previously diagnosed asthma, so these patients are likely to already have knowledge of identifying triggers, controlling and managing their symptoms, thus had better outcomes than those with OA [13]. This exemplifies that asthma education for patients improves management, control and outcomes [63], so many current knowledge translation strategies have attempted to improve education by creating educational tools and management plans [52–58]. However, these strategies only benefit patients who are diagnosed with asthma. There is a gap in knowledge translation strategies to increase awareness of WRA in individuals who have no previous experience with asthma or no knowledge of how their workplace could cause it. Without this knowledge, workers could be at risk of being exposed to triggers for years, thus education should focus on educating workers well before potential exposure to improve awareness and subsequent reporting of WRA.

Another important factor contributing to under-reporting of WRA by patients is the fear of stigma from co-workers and employers and potential loss of work and income which can lead to the healthy worker effect [4, 13, 23–26]. Therefore the lack of awareness of employers is another important gap leading to a delayed diagnosis. While few workplaces have health and safety options [13], clinics that had occupational hygienists and patient representatives had patients with improved quality of life [40]. If more occupational specialists or health and safety options could be provided, then workers might feel more comfortable talking about their symptoms and ultimately reporting and diagnosing their WRA earlier. Additionally, poor relationships with employers also contribute to under-reporting of WRA symptoms [22]. Therefore, education of employers in the signs and symptoms of WRA is necessary to improve their awareness of potential causes. This would not only reduce stigma but would potentially reduce the healthy worker effect. Interestingly, while there are some examples of prevention programs for WRA in some industries [49, 50], beyond this few knowledge translation strategies were found specifically targeting employer attitude and knowledge of WRA or the workplace itself.

Both primary care physicians and specialists have difficulty identifying WRA, taking occupational histories, and communicating the potential relationship between the workplace and asthma to their patients [5, 25, 32, 33, 39]. This, as well as lack access to specialists and failure of using objective measures contribute to the under-reporting and delay in diagnosis of WRA and is indicative of a gap in education and strategy for improving preventive or screening measures for clinicians [32, 35]. Current knowledge translation strategies for healthcare practitioners have involved disseminating guidelines for clinicians to easily apply in practice [39]. Strategies have involved standardizing asthma care plans or pathways for the convenience of the clinician and for education of the patient [59].These help with asthma management or controlling or identifying exacerbations to prevent an attack. Similar to patient knowledge translation strategies, these focus on helping clinicians and patients once a diagnosis has been given. Clinicians could benefit from strategies to improve their awareness of potential WRA and screening for WRA in clinic, especially primary care physicians since they can be the first physician seen by a patient with asthma symptoms [13]. The WRASQ(L) and the OASYS-11 are both tools that are working to help this issue, but since they both still require further validation, the gap in screening and awareness is evident [61, 62].

To improve awareness and reporting of WRA, there should be an increase in education of both employees and employers. If possible, occupational hygenists should be more accessible for workers and clinicians in workplaces and/or clinics, to facilitate WRA awareness for prevention, and screening. Increased employer education and open communication with their employees, especially in high-risk industries is recommended as this will hopefully enable an open conversation between employees and employers and decrease any stigma that might have been associated with it. Improvements are needed in knowledge translation of asthma research moving forward. It is recommended that future knowledge translation strategies focus on improving screening of the workplace and screening in primary care settings to identify potential WRA cases. Particularly, the development and implementation of a validated tool for physicians to use for screening, which would not only improve their awareness and reporting of WRA but would reduce the time to diagnosis, may be a successful knowledge translation strategy . Finally, it is recommended that studies involve the public, since communities prioritize meaningful outcomes from research [45] (Table 1).

## Conclusion

A 4-year delay is seen in the diagnosis of WRA [13]. Key reasons for this relate to a gaps in awareness of the disease by physicians, employers and patients, and under-reporting of the disease by physicians and patients. These gaps in awareness arise from poor knowledge and education of patients and employers on triggers for asthma, and lack of awareness of workplace-symptom relationships, discussion of occupational history by physicians. This lack of awareness contributes to the gap in reporting in patients, as well as stigma from the workplace. Lack access to objective tests, specialists and time for contribute to under-reporting by physicians. Current knowledge translation strategies for asthma research focus on improving education and management of the disease through guidelines, information databases or brochures. However, these strategies focus on improving outcomes once a diagnosis has been made, and the screening process and awareness of this disease has yet to improve. Therefore, there is a need to develop and implement strategies that improve the awareness and detection of WRA. Areas of focus should include accurate diagnosis of asthma, screening for possible WRA at the primary care level, and education of employers and workers who work in areas of potential exposure to prevent development and exacerbations of WRA.

## Data Availability

Not applicable

## Abbreviations

CWHSP: Coal Worker’s Health Surveillance Program
EDACP: Emergency Department Asthma Care Pathway
KTA: Knowledge to Action
OA: Occupational asthma
OASQ-11: Occupational Asthma Screening Questionnaire-11 Items
OSHA: Occupational Safety and Health Administration
PCAPP: Primary Care Asthma Pilot Project
PEF: Peak expiratory flow
WAA: Work-aggravated asthma
WEA: Work-exacerbated asthma
WRA: Work-related asthma
WRASQ(L): Work-related Asthma Screening Questionnaire (Long Version)
